# Optimized Calibration Procedure of the Damage Parameters of 6082-T6 Sheets

**DOI:** 10.3390/ma11020248

**Published:** 2018-02-06

**Authors:** Fan Xu, Hao Zhang, Shengdun Zhao, Chao Chen, Miao Cao, Wei Chen

**Affiliations:** 1 School of Mechanical Engineering, Jiangsu University, Xuefu Road 301, Zhenjiang 212000, China; professorjd@sina.com; 2 Department of Materials Engineering, KU Leuven, Campus Gent, Gebroeders De Smetstraat 1, 9000 Gent, Belgium; zhanghaosteven@163.com; 3 School of Mechanical Engineering, Xi’an Jiaotong University, Xianning West Road 28, Xi’an 710049, China; sdzhao@mail.xjtu.edu.cn (S.Z.); profchenchao@163.com (C.C.); nmggood@163.com (C.M.)

**Keywords:** shear damage parameter, the initial void volume fraction, orthogonal analysis

## Abstract

The constitutive equation of AA6082-T6 sheets is investigated by an extended Rousselier damage model. Previous research is mainly comprised of single-pull test specimens, notched tensile specimens, tensile specimens with a hole, and shear specimens. To decrease the natural material errors, a new specimen is used. In this paper, a clinched joint is regarded as a specimen to calibrate the initial void volume fraction $k_{\omega }$ and the shear damage parameter $f_{0}$ by using the orthogonal analysis method, which can reduce the simulation times and accuracy. It also reveals that the initial void volume fraction $f_{0}$ affects the void volume fraction in the neck and the groove of the clinched joint, and the shear damage parameter $k_{\omega }$ affects just the void volume fraction f in the neck of the clinched joint. It checks the force-displacement curve, shape of the clinched joint, and the fracture location, and approves that these damage parameters can describe the deformation process, fracture location, and shape of the clinched joint.

## 1. Introduction

The request to reduce fuel consumption, emissions, and weight in the automotive industry has previously led to the search for a new joining technique and material capability. The mechanical durability of the joints depends on several parameters, including the specific joining technique, the nature of assembled materials, and the corrosion stability of the constituents. AA6082-T6 sheets are widely used in the automotive industry as a basis for lightweight alternatives to classical designs with mild and high-strength-low alloyed steels. Dadbakhsh [1] explored the AA6082-T6 sheet and studied the alloy strengthening because of pre- and post-ECAP (Equal channel angular pressing) aging treatment. It showed that aging before and after ECAP processing was an effective method for strengthening of the alloy. Chen [2] suggested that the stress-strain behavior of extruded AA6xxx and AA7xxx aluminum alloys in T6 temper should be studied at a wide range of strain rates. The AA6xxx alloys were found to exhibit an insignificant rate sensitivity in the stress-strain behavior. 

Some papers focused on the deformation of the alloy sheet to discuss the application in some fields, such as the deep drawing, cold welding process, tensile tension, and mechanical connection. A great deal of the experimental research will greatly increase the cost of the production, and the selection of a suitable constitutive equation to obtain the parameters is impossible. The FE (finite element) simulation analysis is an essential method in studying the deformation process, as it can obtain some parameters without any experimentation. The success of the simulation model depends upon the capacity of the constitutive models and respective material parameters to accurately reproduce the mechanical behavior. However, the macro-constitutive or meso-constitutive model is required to be used. The material constitutive models were used to describe the deformation and failure processes of the metal sheet. At present, some researchers focus on some damage models, such as the extension GTN and Rousselier models.

Some failure behavior occurs during the deformation process and, commonly, ductile failure is a major failure behavior. Ductile damage is strongly linked with the plastic behavior of the material, and failure occurs along with the combined mechanism of plastic strain localization, damage nucleation, growth, and coalescence. Moreover, several ductile damage models are available in the literature. Slimane [3] was devoted to the numerical simulation of axisymmetric notched specimens to study the phenomenon of nucleation by the GTN model. Dunand and Mohr [4] evaluated the predictive capabilities of the shear-modified Gurson and Mohr–Coulomb fracture models. The shear-modified Gurson model is based on the assumption that void growth is the governing mechanism, whereas, the Mohr–Coulomb model is based on the assumption that the initiation of fracture is determined by the critical stress state. Klingbeil [5] used the evolution strategy to help identify the parameters of Gurson model for the high-strength steel 10MnMoNi5-5 with respect to cyclic loading at two temperatures. Ayatollahi [6] used the Gurson–Tvergaard–Needleman model to describing the ductile damage in the ferritic matrix. Simultaneously, the numerical results that were obtained from the randomly extruded 3D model showed a better agreement with the experimental results than those obtained from the 2D model or the evenly-extruded 3D model. These models ignored the shear stress due to the failure behavior under the deforming process; then some researchers modified the original damage model. Xue [7] explored an extension of the Gurson model that incorporates damage development in shear is used to describe the tension-torsion test fracture data. Two damage parameters, including the initial effective void volume fraction and the shear damage parameter were calibrated by notched round tensile bars and shear specimens. Xu and Zhao [8,9] discussed some failure models, including the abnormal failure and the normal failure, and studied the deformation mechanism with the modified GTN model and Rousselier models. However, the accuracy of these models rely directly on the accurate identification of the parameters for each material. Zhao [10] extended an original GTN model by combining the damage mechanic concept and porous plasticity model. Enakoutsa [11] has developed a new methodology to describe the damage in the Gurson model for porous ductile materials. Achouri [12] proposed a new extension of the Gurson damage model that is believed to predict the ductile fracture under shear dominated loads. Simultaneously, the extended Gurson damage model is tested and applied in the punching process to compare its predictive ability with the original approach. 

Xue [13] studied that the sensitivity of the damage parameters, and used several key modelling techniques including the friction coefficient identification and digital image correlation were performed. Guo [14] explained the failure behavior by the void theory, explored a modified Rousselier model, and introduced a calibration procedure of the damage parameters. This modified damage model can describe the evolution of the fracture with the initial void volume fraction and the shear damage parameter. Coppieters et al. [15] used a modified Rousselier damage model [14] to describe the fracture behavior in the bottom of the clinched joint under overload. Lambiase and Di Ilio [16] explored a numerical model that describes the evolution of ductile damage in predicting the onset of fracture during the clinch joining of thin aluminum AA6082-T6 sheets. They used instrumented punch-out tests to calibrate the ductile damage parameters to increase the reliability and robustness. Roux and Bouchard [17] focused on the identification method by inverse analysis of Lemaitre damage model parameters. Basically, the identification of damage parameters is an important task. M.V. Jr [18] addresses the identification of material parameters for Gurson and Lemaitre constitutive models for a low-alloy steel based on the hybrid global-local optimization technique. The Lemaitre-type material can provide a somewhat better approximation of the experimental data than the Gurson model. Yanshan Luo [19] explored a ductile fracture model to describe shear fracture of sheet metals from shear to balanced biaxial tension via uniaxial and plane strain tension. The fracture loci of AA6082 T6 are also constructed by the MMC3, DF2012, and DF2014 criteria for the purpose of comparison. These criteria are implemented into numerical prediction of ductile fracture in shear, uniaxial tension, plane strain tension, and balanced biaxial tension.

These damage models were applied to describe the deform process of metal material, such as deep drawing, cold welding process, tensile tension, and mechanical connection. Commonly, it is difficult to obtain a uniform constitutive equation for all materials. At present, the modified GTN and modified Rousselier damage models also introduced the shear damage parameter to describe the failure behavior. However, previous studies cannot describe the fracture in the groove of the clinched joint. In Xu’s [8] paper, a clinched joint should be divided into four regions, including the no deforming region, bending region, compress region, and tensile region. In order to obtain accurate damage parameters, the clinched joint was regarded as a specimen to calibrate the damage parameters. 

The present study uses data from the uniaxial tensile specimen, and the clinched joint test is also used to identify the intrinsic uniaxial stress-strain behavior, the initial void volume fraction, and the shear damage parameter. The paper is organized and closely follows the steps in the calibration agreement. In Section 2, the determination of the hardening behavior from the tensile tests is made. In Section 3, the determination of the initial void volume fraction and the shear damage parameter from the clinched joint by the orthogonal test is made. Simultaneously, a validation of the shear damage parameter and the initial void volume fraction of the extended Rousselier damage model [9] is performed. In Section 4, the applicability of the calibrated constitutive model to the clinched joint failure mode id discussed as one illustration. Possible variations in the identification agreement for other ductile materials are also discussed. 

## 2. Calibration of the Hardening Behavior

**Figure 1:**
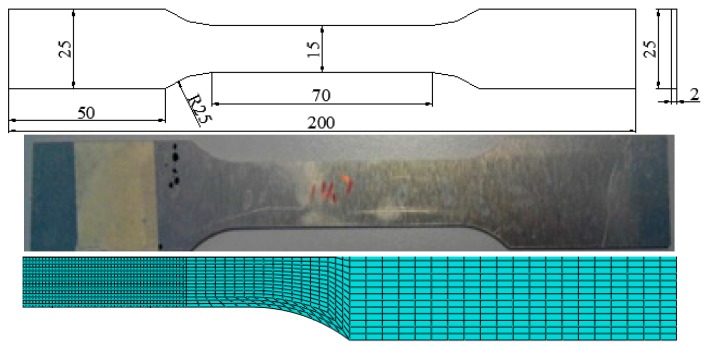
The specimens for the uniaxial tensile test.

The AA6082-T6 sheets were cut into tensile test specimens. The samples for the tensile test were 200 mm long and 25 mm wide, as shown in Figure 1. The mesh size in the densest area is 100 μm × 100 μm. The mechanical response of the undamaged materials was obtained from quasi-static uniaxial tensile tests on the specimen (Figure 1) with elastic–plastic finite element computations. The test geometry and finite elements are shown in Figure 1. AA6082-T6 with a nominal chemical composition in Table 1 was the candidate for the micro-indentation test to inspect the fracture toughness. The micro-indentation specimens were carefully ground with sandpaper. The specimens were polished with 5th and 1th grit diamonds to a mirror finish. The specimen tests were conducted under quasi-static loading, whereas all simulations were performed with the dynamic code in ABAQUS/Explicit [9]. To minimize the inertial effects and efficiently simulate the quasi-static tests in the explicit code, a preliminary series of calculations with different fixed and applied loading rates was performed for each test configuration. At some loading rates, the simulations converge to a quasi-static limit as the rates decrease. The loading rate was then employed in all subsequent calculations. 

In the paper by Luo [19], the experimental results show a strong dependency of the strain to fracture the material orientation with respect to the loading direction. The use of the isotropic modified Mohr–Coulomb stress state weighting function in this anisotropic fracture modeling framework provides accurate predictions of the onset of fracture for all thirteen fracture experiments. Material strain-rate dependence is ignored in the present computations. Each test was conducted five times, and the average values were calculated to eliminate the error.

**Figure 2:**
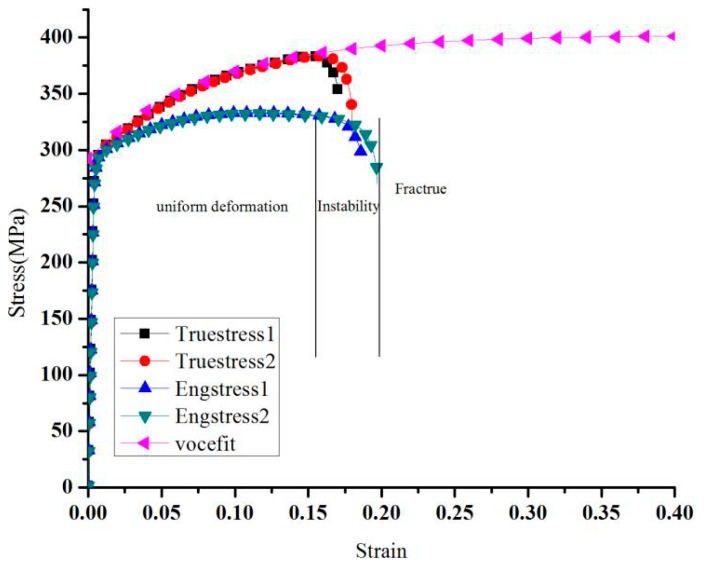
Fitting curve for AA6082-T6.

According to the tensile test, the engineering stress and strain curve can be obtained. Simultaneously, the true stress and true strain can be obtained with the equations. In Figure 2, better hardening behavior was shown for a better long space; therefore, the stress strain curve for a long space is difficult to check with the tensile test. In this paper, the Voce equation is used to fit the performance of the AA6082-T6 sheet with a thickness of 2 mm. The simulation results and the experiment results of the stress-strain curves are shown in Figure 2. Basically, prior to necking, the true strain is given by $\varepsilon_{T}=\ln(1+\varepsilon_{N})$, and the true stress is given by $\sigma_{T}=\sigma_{N}(1+\varepsilon_{N})$. To deduce the true response in the post-necking regime, computations were performed with an assumed form of the stress-strain relation and matching the predicted force-displacement curves with those obtained experimentally. 

As shown in Figure 2, this hardening equation cannot accurately describe the hardening process of the AA6082-T6 sheet. The VOCE hardening equation can describe the part before the uniform deformation, and the part after the uniform deformation needs a new hardening equation to fit. Consequently, in this paper, the VOCE hardening behavior is modified. Before the uniform deformation, the hardening model VOCE was used. After the uniform deformation, the shear band was shown, and the normal hardening model cannot be used. However, the material sheet should still be hardened, and the hardening curve is unknown. Coppieters et al. [20] presented a method to obtain the hardening equation with Digital Image Correlation (DIC). Luo [21] discussed an optimized method comprehension for all experimental results and adjusted the hardening curve to save the accordance between the simulation and experimental results according to the displacement and the force curve. 

This study agreed with the paper by Xue [22]. The material was unstable when the strain value was 0.14. Actually, the maximum equivalent strain should be 0.1522 when the deformation of material reaches instability. Thus, the two hardening equations can be described in the AA6082-T6 sheet: the hardening behavior and the mixed hardening behavior:

(1) $$\sigma =\{\begin{array}{c} A-(A-B)\exp(-C\varepsilon )\begin{array}{cc} & \mathrm{voce} \end{array}\\ \sigma^{peak}{\left(\varepsilon /\varepsilon^{peak}\right)}^{n}\begin{array}{cccc} & & & \mathrm{xue} \end{array} \end{array}$$

where *A* = 402.05, *B* = 292.56, *C* = 12.26, $\sigma^{peak}=385.5326$, and $\varepsilon^{peak}=0.1522$.

(2) $$\sigma =\{\begin{array}{c} 402.05-(402.05-292.56)\times \exp(-12.26\times \varepsilon )\begin{array}{cc} & \varepsilon \leq \end{array}0.1522\\ 383.5326\times {\left(\varepsilon /0.1522\right)}^{n}\begin{array}{cccc} & & & \varepsilon >0.1522 \end{array} \end{array}$$

**Figure 3:**
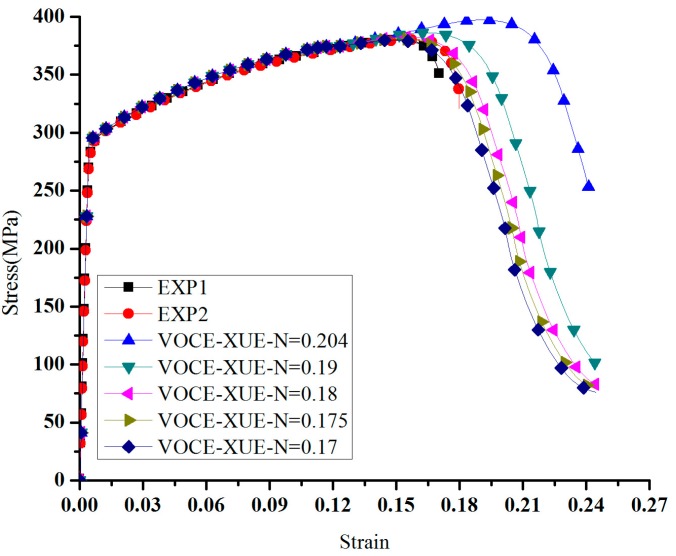
The hardening behavior for VOCE-XUE.

A preliminary estimate of the strain hardening exponent *n* was obtained by fitting. A series of finite element computations was performed to ascertain the full nominal tensile stress-strain curve with a range of *n* values. As shown in Figure 3, the results for *n* = 0.18 accurately replicate the experimental measurements up to the onset of rupture. When the strain is smaller 0.1522, the voce hardening equation was used. When the strain is larger than 0.1522, Xue’s equation was used to describe the hardening behavior. In other words, Xue’s equation will be used after instability.

All the simulation results show that the mixed hardening equation can accurately be described by the hardening process of the AA6082-T6 sheet with 2 mm when the hardening index *n* is equal to 0.18. Other than $f_{0}$ and $k_{\omega }$, the basic parameters characterizing the constitutive model that are used in all simulation in this paper are the following: E=70188MPa, ν=0.33, $f_{c}=0.2$, n=0.18. 

In Guo’s paper [14], the yield potential is written as Equation (3), so it is a coupled constitutive equation in which the damage accumulation and hydrostatic stress are incorporated:

(3) $$\phi =\frac{\sigma_{eq}}{\rho }-R(p)+Df\sigma_{1}\exp(\frac{\sigma_{m}}{\rho \sigma_{1}})=0$$

where $\sigma_{m}$ is hydrostatic stress, $\sigma_{eq}={\left(3\underline{\underline{\sigma_{d}}}:\underline{\underline{\sigma_{d}}}/2\right)}^{1/2}$ is the von Mises equivalent stress, $\rho =(1-f)/(1-f_{0})$ is the relative density, f is the damage variable or void volume fraction, $f_{0}$ is the initial void volume fraction in the material, R(p) is the hardening function of the material, $p={\left(2\underline{\underline{\varepsilon_{d}^{p}}}:\underline{\underline{\varepsilon_{d}^{p}}}/3\right)}^{1/2}$ is the equivalent plastic strain, D and $\sigma_{1}$ are Rousselier material constants:

(4) $$\overset{\cdot }{f}=[Df(1-f)\exp(\frac{\sigma_{m}}{\rho \sigma_{1}})+k_{\omega }f\omega (\underline{\underline{\sigma }})]\overset{\cdot }{p}$$

Here, $k_{\omega }$ is the shear damage coefficient, which sets the magnitude of the void coalescence rate in shear deformation on the basis of Junhang Guo et al. [14]. And the invariant measure $\omega (\underline{\underline{\sigma }})$ is given by:

(5) $$\omega (\sigma )=1-\xi^{2}=1-{\left(\frac{27J_{3}}{2\sigma_{eq}^{3}}\right)}^{2}$$

(6) $$J_{3}=\det({\underline{\underline{\sigma }}}_{d})=(\sigma_{1}-\sigma_{m})(\sigma_{2}-\sigma_{m})(\sigma_{3}-\sigma_{m})$$

where $J_{3}$ is the third stress invariant of the deviatoric stress tensor ${\underline{\underline{\sigma }}}_{d}$ ($\underline{\underline{\sigma_{d}}}$ is the deviatoric stress tensor); $\sigma_{1}$, $\sigma_{2}$, and $\sigma_{3}$ are the principal stresses of the stress tensor $\underline{\underline{\sigma }}$ and are assumed to be ordered as $\sigma_{1}\geq \sigma_{2}\geq \sigma_{3}$. The non-dimensional metric in Equation (5) lies in the range 0≤ω≤1 to discriminate between axisymmetric and shear-dominated stress states. For all axisymmetric stress states, ω=0. For all states comprised of a pure shear stress plus a hydrostatic contribution, ω=1. With $f_{0}$ as the initial void volume fraction, the analytical solution can be derived as:

(7) $$f=\frac{(D+k_{\omega }\omega (\underline{\underline{\sigma }}))f_{0}}{(D+k_{\omega }\omega (\underline{\underline{\sigma }})-Df_{0})e^{-(D+k_{\omega }\omega (\underline{\underline{\sigma }}))p}+Df_{0}}$$

Then for the shear stress state, the solution can be particularized with D=2 (Junhang Guo [14]), and $\omega (\underline{\underline{\sigma }})=1$ as:

(8) $$f=\frac{(2+k_{\omega })f_{0}}{(2+k_{\omega }-2f_{0})e^{-(2+k_{\omega })p}+2f_{0}}$$

From Equation (8), it can be seen that the damage coefficients $f_{0}$ and $k_{\omega }$ need be calibrated. In this paper, the damage coefficients of the material were identified by an inverse method. It described the clinching process to calibrate the extended Rousselier damage model.

## 3. Calibration of the Damage Parameters $f_{0}$ and $k_{\omega }$

### 3.1. 3D Model

**Figure 4:**
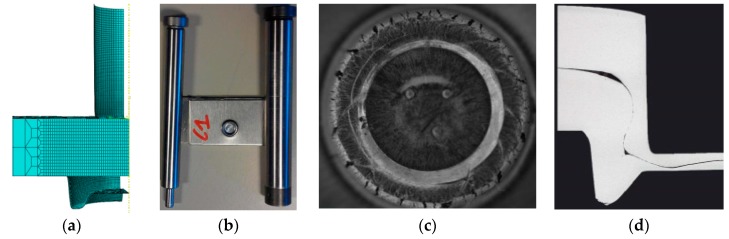
Simulation model, experiment tools and specimen. (**a**) 3D model; (**b**) specimens and tools; (**c**) the clinched joint with a fracture; and (**d**) the crack happening in the groove of the clinched joint.

Based on the previous discussion, the use of the failure characteristics during the clinching process calibrated the shear damage parameter and the initial void volume fraction. In the first section, a new specimen was proposed to optimize the calibration of damage parameters. Figure 4 shows a 3D model of the experimental sample with the groove of the clinched joint and in the neck of the clinched joint. Figure 4a is the 3D model based on the paper by Xu [8]. The mesh was divided into three parts: the intensive, transition, and loose regions. The symmetric boundary condition is used and one-quarter geometry is modeled. To improve the computational efficiency, only the material in the clinched joint region is modeled using the modified Rousselier model. Outside this zone the plate is modeled using the standard von Mises plasticity model provided by ABAQUS/Explicit. The minimum element size is 100 μm × 100 μm × 100 μm. Adaptive meshing is employed to avoid element distortion in the large localized shear deformation in the simulation. Figure 4b shows the tools and the clinched joint, wherein the die is closed. Figure 4c shows a specimen where the fracture just happened in the groove of the clinched joint, but in the neck of the clinched joint. Figure 4d shows that the cross-section of the clinched joint, including all geometrical parameters of the clinched joint, is simultaneously used to find the obvious fracture that happened in the groove and neck of the clinched joint.

The fracture obviously happened in the groove of the clinched joint but not in the neck of the said joint in Figure 4. The tensile stress causes a crack in the groove of the clinched joint. In Xue’s paper [7], the groove region belongs to the tensile deformation region. In the previous discussion, Xu [8] proposed that the shear stress and tensile stress cause the crack in the neck of the clinched joint. If the crack happens in the neck of the clinched joint, the shear damage parameter should be controlled. During the tensile test process, the reduced thickness of the sample (12%) is smaller than the thickness (84%) of the neck of the clinched joint. When the bottom thickness reached at 0.56 mm, the reduced ratio thickness is 86%. The crack happened in the groove of the clinched joint. Local tensile deformation leads to the crack that happens in the groove of the clinched joint. The local deformation region reached the yield limitation, and then the crack starts at this point. Therefore, the fracture can be controlled in the groove. 

### 3.2. Orthogonal Analysis

To reduce the fitting time and evaluate the computation results, an effective design method is needed [23]. Several different factors and levels need to be considered when optimizing the parameters during the clinching joint. The purpose of the optimization of parameters during the clinched joint is to get the damage parameters of the Rousselier damage model. Orthogonal design is a kind of design method that is mainly used to study multiple factors and multiple levels. This design method is uniformly dispersed, neat, and comparable, making each design highly representative. The selection of the representative points from the comprehensive design can fully reflect the impact of different levels of each factor on the design result that takes less time. Oudjene and Ben-Ayed [24] used the Taguchi’s experiment design method that is used to investigate the effects of geometry tools on the clinch joint resistance as well as on its shape. Chen [25] designed a plan to optimize the shape of the rivet to reduce the protrusion height and increase the strength of clinched joint. Wen [26] presented a response surface optimization of the clinching tools.

During the clinched process, the clinched joints with fracture zone in the groove of the clinched joint are obtained. The previous research mainly discussed how to get some qualified samples to avoid the failure samples. In the discussion, some ideas are discussed. A lot of experiments show that the surface stage will affect the quality of the clinched joint; thus, in our research, the grease is used to avoid the damage that comes from the surface stage. The friction between tools and sheets slightly influences the intensity of the punch force and the distribution of deformation between the upper and lower sheets. This tendency is even greater with respect to metal hardening. 

Since the fracture just happened, in the groove of the clinched joint during the clinched process, it must be ensured that there will be no cracks in the neck of the clinched joint. In the paper by Xu [8], the clinched joint is divided into four parts, including no deformation zone, tensile deformation zone, shear deformation, and bending deformation zone. Zhao [9] presented that the shear damage will happen in the neck of the clinching joint. Therefore, the assumption is that the shear deformation region check the shear damage parameter $k_{\omega }$ and the tensile deformation region and validate the initial void volume fraction $f_{0}$ has been made. In other words, the shear damage parameter $k_{\omega }$ and the initial void volume fraction $f_{0}$ affect the quality of the clinched joint. The purpose functions are $f_{1}$, $f_{2}$, and F. The function $f_{1}$ expresses the void volume fraction when crack happens in the neck of the clinched joint, whereas $f_{2}$ expresses the void volume fraction when crack happens in the groove of the clinched joint. The function F expresses the deformed force from the experimental results when the bottom thickness of the clinched joint is 0.56 mm. 

Several factors and levels have been investigated. In order to facilitate research, it need to construct a suitable orthogonal table [23]. In Section 2, it introduces the function of the void volume fraction and discusses the relationship between the shear damage parameter and the initial void volume fraction. It has explained that the shear damage parameter and the initial void volume fraction, they are independent parameters. In this paper, the relationship between shear damage parameter and the initial void volume fraction is ignored. The suitable level range will reduce the number of optimization design. Therefore, before the orthogonal analysis, it is necessary to predict the shear damage parameter and the initial void volume fraction, that is, use the specimen shown in Figure 1 to determine a rough range. Finally, six five-level factors are contained in the orthogonal array used for the optimization of parameters during the clinched process as presented in Table 2 (L25(5^6^)). These factors are more helpful in getting the suitable damage parameters during the clinching process. Commonly, lubricant is used between punch and upper sheet, so $\mu_{1}$ is equal to 0. The critical void volume fraction is equal to 0.2 [27]. In fact, four parameters, $\mu_{2}$, $\mu_{3}$, $k_{\omega }$, and $f_{0}$ need be calibrated by this method. $\mu_{1}$ and $f_{c}$ are regarded as fixed constants. Then a group of 25 different parameter combinations should be simulated to find the optimal the damage parameters and the state parameters according to the orthogonal array as shown in Table 3A–F represent the friction coefficient between punch and the upper sheet $\mu_{1}$, the friction coefficient between the upper sheet and the under sheet $\mu_{2}$, the friction coefficient between the die and the under sheet $\mu_{3}$, the shear damage parameter $k_{\omega }$, the initial void volume fraction $f_{0}$, the critical void volume fraction $f_{c}$, respectively. 

**Figure 5:**
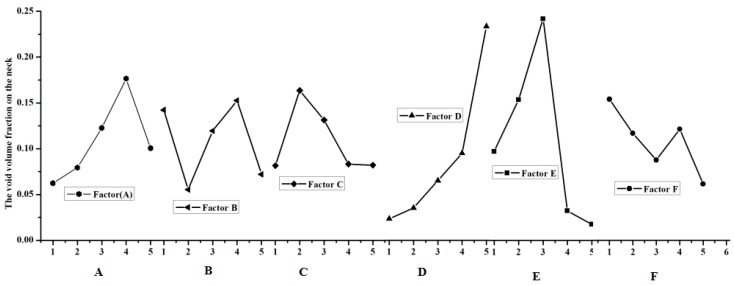
Effect of process parameters on the fracture location on the neck.

**Figure 6:**
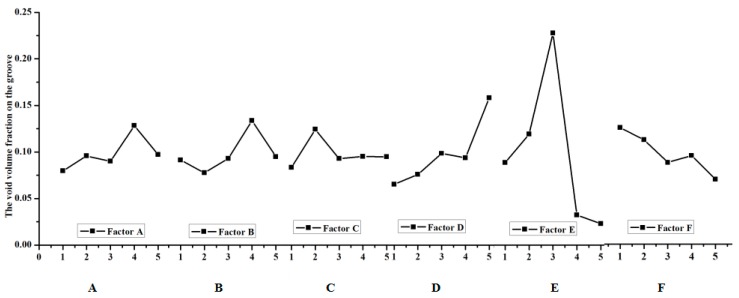
Effect of process parameters on the fracture location on the groove.

**Figure 7:**
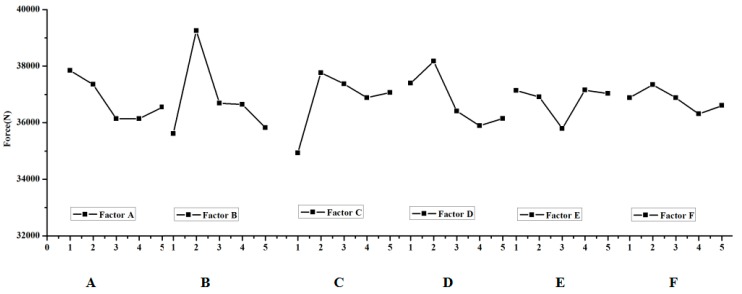
Effect of process parameters on the force during the clinching process.

The extent of the effect in the void volume fraction during the clinching process should be investigate; in other words, a method should be developed on how to get the crack that happens in the groove of the clinched joint. The range analysis was made according to the results of the numerical simulation. The data of the range analysis are shown in Table 4, Table 5 and Table 6. The effect curves of the parameters are shown in Figure 5, Figure 6 and Figure 7. 

The most important factor that affects the fracture location in the neck of the clinching joint is shown in Table 4. K_a_ expresses the sum of test indicators (a expresses level (1, 2, 3, 4, 5)), i expresses level (1, 2, 3, 4, 5), j expresses factor (A, B, C, D, E, F), k_ij_ is equal to K_ij_ divided by 5, rank is equal to the maximum (k_ij_) minus the minimum (k_ij_). Table 4 shows thatextreme difference analysis for the void volume fraction on the neck of the clinched joint. Factor D has an influence on the void volume fraction f on the neck of the clinched joint. D expresses the shear damage parameter $k_{\omega }$. The previous research shows that the shear stress leads to the failure on the neck of the clinched joint. Thus, the orthogonal analysis method can describe the trend. Table 4 shows that the shear damage parameter $k_{\omega }$ has a great influence on the void volume on the neck of the clinched joint; simultaneously, the initial void volume fraction $f_{0}$ also influences the void volume fraction f on the neck of the clinched joint. The shear damage parameter $k_{\omega }$ is the one of the most important factors on the neck of the clinched joint. Except on the shear damage parameter $k_{\omega }$ and the initial void volume fraction $f_{0}$, the shear damage parameter $k_{\omega }$ also has an impact on other factors. Table 5 shows that the initial void volume $f_{0}$ has a signification influence on the void volume fraction f on the groove of the clinched joint. Table 6 shows that the coefficient friction $\mu_{2}$ between the two sheets is one of the most important factors on the force F. The extent in which the factors affect the deform force F is shown in Figure 6, and the effect curves of the factors are also shown in Figure 6. The final optimized results cannot be obtained through extreme difference analysis because the orthogonal analysis’ maximum or minimum value is not a subject value. The orthogonal analysis was used to get the influence trend of the void volume fraction on the different deformation regions (neck and grove regions). The initial void volume fraction $f_{0}$ and the shear damage parameter $k_{\omega }$ also obviously affect the void volume fraction f on the neck of the clinched joint (Figure 5 and Table 4). During the simulation process, the initial void volume fraction $f_{0}$ and the shear damage parameter $k_{\omega }$ must be controlled to ensure that there will be an absence of cracks in the neck of the clinched joint. Apparently, the initial void volume fraction $f_{0}$ affected the void volume fraction f distribution in the groove of the clinched joint as shown in Figure 6 and Table 5. Simultaneously, the factor that influences the deform force is known. 

**Figure 8:**
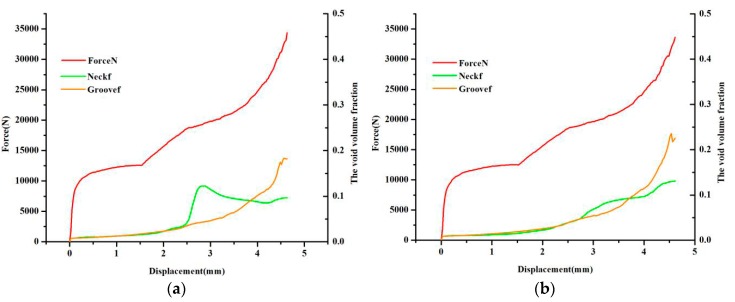
The subject parameters changing. (**a**) The original damage parameter; and (**b**) the optimized damage parameter.

Unlike other studies, in this paper, the orthogonal analysis method was used, but the subject function cannot be considered as the final result. The maximum or minimum value can evaluate the influence trend, but it cannot be considered as the final result. The aim of the paper is to discuss the calibration of the damage parameters for AA6082-T6 sheet with a thickness of 2 mm by the clinching process according to the fracture location. After 25 simulation results, it is easy to find that the simulation of No.10 is close to the experimental result. However, as shown in Figure 8a, no fracture happens in the neck and the groove of the clinched joint. Apparently, the void volume fraction f in the groove of the clinched joint is close to 0.2, whereas the void volume fraction f in the neck of the clinched joint is almost close to 0.1. The subject stage is the fracture in the groove of the clinched joint; thus, according to the above analysis, an investigation must be conducted if the initial void volume fraction meets the fracture location. The second optimized result was shown in Table 7. 

Compared with the two group parameters as shown in Figure 8, the fracture obviously happens in the groove of the clinched joint, but in the neck of the clinched joint, as shown in Figure 8b, there was no crack that was observed anywhere because the void volume fraction f is smaller than the critical void volume fraction $f_{c}$ when the initial void volume fraction $f_{0}$ is 0.009. Evolution of the damage at the neck of the clinched joint, shear damage parameter $k_{\omega }$, and the initial void volume fraction $f_{0}$ lead to the damage. In the groove of the clinched joint, the initial void volume fraction $f_{0}$ leads to the fracture. If the shear damage parameter $k_{\omega }$ was not discussed, the shear stress in the neck of the clinched joint will be neglected. That means an important factor can be ignored, and then there is a greater difference between the simulation results and the experiment results. It was observed that the void volume fraction of tracked nodes normally ranged from 0.001 to 0.01. The shear damage parameter $k_{\omega }$ normally ranged from 0.5 to 1. 

**Figure 9:**
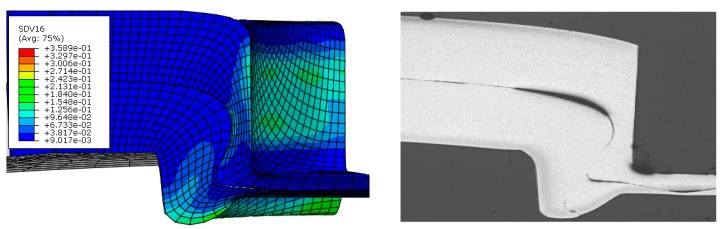
The damage parameter (SDV16).

The simulation result for the damage parameter of the initial void volume fraction $f_{0}$ distribution on the clinched joint is shown in Figure 9. The maximum value of SDV16 (the void volume fraction) is shown in the groove of the clinched joint. Compared with the experimental results, the fracture that happens in the groove of the clinched joint is obviously noted. Thus, this model is known to describe the failure location; in other words, these damage parameters can accurately describe the performance of the AA6082 sheet with a thickness of 2 mm during the clinched process. 

**Figure 10:**
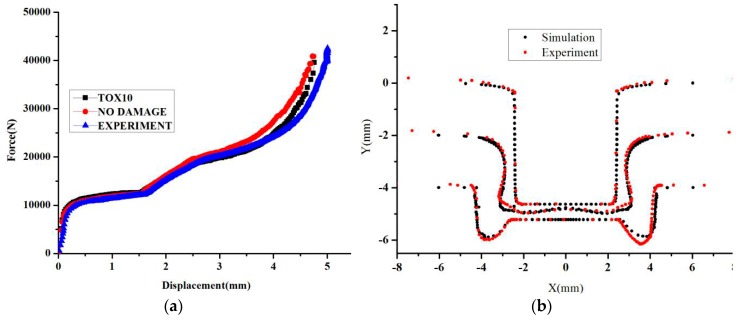
The displacement-load curve and the shape of the clinched joint. (**a**) Deform force; and (**b**) The shape of the clinched joint.

Figure 10 shows that the displacement-force curve and the shape of the clinched joint is shown in Figure 10. The comparison experiment results and simulation results, including undamaged model and Rousselier damage model, are shown in Figure 10a. The undamaged model was used to describe the clinching process, and a larger difference between the simulation results and the experiment results is noted. When the Rousselier damage model was used to describe the clinching process, a good consistency was shown in Figure 10a. Therefore, this model is suitable for describing the clinching process and explaining the performance of the AA6082-T6 sheet with 2 mm thickness. In Figure 10b, the shape from simulation is known to be in accordance with the shape from the experiment. In summary, this model can describe the clinching process, including the force-displacement curve, the crack location, and the clinched joint shape. Thus, the orthogonal analysis method is also shown to obtain the suitable damage parameters, and the damage parameters can be obtained by the clinching process.

## 4. Concluding Remarks

The present work describes a methodology to identify the Rousselier damage model parameters with the clinched joint experimental–numerical approach. A clinched joint test program includes experiments for both tension- and shear-dominated loading. Based on the paper by Xu et al. [8], the neck of the clinched joint can check the shear damage parameter, whereas the groove of the clinched joint can simultaneously validate the void volume fraction. The main conclusions of this work as follows:

(1) The constructs demonstrate a segment-hardening behavior, and this hardening behavior can be described by the post-necking hardening phenomena. When the equivalent strain $\varepsilon_{eq}$ is less than 0.1522, the Voce function is used as the hardening equation. When the equivalent strain $\varepsilon_{eq}$ is more than 0.1522, the equation fits the hardening behavior. Finally, the hardening equation at room temperature for AA6082-T6 sheet is determined.
(2) To calibrate the shear damage parameter and the initial void volume fraction simultaneously, a clinched joint is used. The reasons for the use of this sample are analyzed. Orthogonal analysis is used to calibrate the parameters during the clinching process and to build five of the six-level factors. According to the fracture that happens in the groove of the clinching joint, the void volume fraction f is compared with the critical void volume fracture $f_{c}$ to obtain the purpose function. The factors $f_{1}$, $f_{2}$ and F are used to evaluate the results. Simultaneously, orthogonal analysis can be described by the sensitivity of the damage parameters on the different deformation regions. The initial void volume fraction $f_{0}$ affects the void volume fraction in the neck and the groove of the clinched joint, and the shear damage parameter $k_{\omega }$ affects just the void volume fraction f in the neck of the clinched joint. The second optimization obtained the better damage parameters.
(3) The good agreement of the numerical predictions with all experimentally-measured force-displacement curves validates the extended modified Rousselier model. Simultaneously, the experimental results on the force-displacement curve, the shape of the clinched joint, and the fracture location are compared with the simulation results. It proved that these damage parameters can describe the deformation process, fracture location, and shape of the clinched joint. These parameters can describe the performance of AA6082-T6 sheet with 2 mm thickness.

## Figures and Tables

**Table 1:** Chemical compositions of Al6082-T6 sheets.

Element	Mn	Fe	Mg	Si	Cu	Zn	Ti	Cr	Al
Mass contents (%)	0.4–1	0–0.5	0.6–1.2	0.7–1.3	0–0.1	0–0.2	0–0.1	0–0.25	Balance

**Table 2:** The parameters and the levels.

	$\mu_{1}$	$\mu_{2}$	$\mu_{3}$	$k_{\omega }$	$f_{0}$	$f_{c}$
1	0	0	0	0.3	0.003	0.2
2	0	0.1	0.1	0.5	0.005	0.2
3	0	0.2	0.2	0.8	0.008	0.2
4	0	0.3	0.3	1	0.0008	0.2
5	0	0.4	0.4	1.5	0.0004	0.2

**Table 3:** The results of orthogonal design.

	A	B	C	D	E	F	$f_{1}$	$f_{2}$	F
1	1	1	1	1	1	1	0.02345	0.0430	35,781.4
2	1	2	2	2	2	2	0.05204	0.09818	43,265.3
3	1	3	3	3	3	3	0.17883	0.18898	36,964.5
4	1	4	4	4	4	4	0.02379	0.03861	36,703.2
5	1	5	5	5	5	5	0.03340	0.0296	36,496
6	2	1	2	3	4	5	0.01523	0.02080	36,875.6
7	2	2	3	4	5	1	0.00951	0.01793	39,763.5
8	2	3	4	5	1	2	0.2340	0.15229	37,530.6
9	2	4	5	1	2	3	0.04127	0.10554	38,221.3
10	2	5	1	2	3	4	0.09661	0.18200	34,368
11	3	1	3	5	2	4	0.40926	0.15579	34,470.4
12	3	2	4	1	3	5	0.05097	0.13477	38,049.9
13	3	3	5	2	4	1	0.01112	0.02119	38,079.1
14	3	4	1	3	5	2	0.02725	0.04993	34,480.9
15	3	5	2	4	1	3	0.11517	0.08876	35,625.3
16	4	1	4	2	5	3	0.00785	0.01052	36,701.5
17	4	2	5	3	1	4	0.06851	0.09250	38,283.5
18	4	3	1	4	2	5	0.16488	0.09662	33,151.3
19	4	4	2	5	3	1	0.62655	0.40681	35,349.6
20	4	5	3	1	4	2	0.01539	0.03488	37,204.8
21	5	1	5	4	3	2	0.25578	0.22649	34,241
22	5	2	1	5	4	3	0.09505	0.04571	36,898.5
23	5	3	2	1	5	4	0.00874	0.00782	37,726.9
24	5	4	3	2	1	5	0.04367	0.06695	38,465.2
25	5	5	4	3	2	1	0.09955	0.13950	35,443

**Table 4:** Extreme difference analysis for the void volume fraction on the neck of the clinched joint.

	A	B	C	D	E	F
K_1_	0.311510	0.711570	0.407240	0.139820	0.484800	0.770180
K_2_	0.396620	0.276080	0.817730	0.211290	0.767000	0.584460
K_3_	0.613770	0.597570	0.656660	0.389370	1.208740	0.438170
K_4_	0.883180	0.762630	0.416160	0.569130	0.160580	0.606910
K_5_	0.502790	0.360120	0.410080	1.398260	0.086750	0.308150
k_1_	0.062302	0.142314	0.081448	0.027961	0.096960	0.154036
k_2_	0.079324	0.055216	0.163546	0.042258	0.153400	0.116892
k_3_	0.122754	0.119514	0.131332	0.077874	0.241748	0.087634
k_4_	0.176636	0.152526	0.083232	0.113826	0.032116	0.121382
k_5_	0.100558	0.072024	0.082016	0.279652	0.017350	0.061630
Rank1	0.114334	0.097310	0.082098	0.251691	0.224398	0.092406
R	3	4	6	1	2	5

**Table 5:** Extreme difference analysis for the void volume fraction in the groove of the clinched joint.

	A	B	C	D	E	F
K_1_	0.398370	0.456600	0.417260	0.326010	0.443500	0.628430
K_2_	0.478560	0.389090	0.622370	0.378840	0.595630	0.561770
K_3_	0.450440	0.464530	0.464530	0.491710	1.139050	0.439510
K_4_	0.641330	0.667840	0.475690	0.468410	0.161190	0.476720
K_5_	0.486470	0.474740	0.475320	0.790200	0.115800	0.348470
k_1_	0.079674	0.091320	0.083452	0.065202	0.088700	0.125686
k_2_	0.095712	0.077818	0.124474	0.075768	0.119126	0.112354
k_3_	0.090088	0.092906	0.092906	0.098342	0.227810	0.087902
k_4_	0.128266	0.133568	0.095138	0.093682	0.032238	0.095344
k_5_	0.097294	0.094948	0.095064	0.158040	0.023160	0.069748
Rank2	0.048592	0.055750	0.041022	0.092838	0.204560	0.055938
R	5	4	6	2	1	3

**Table 6:** Extreme difference analysis for the deform force.

	A	B	C	D	E	F
K_1_	189,210.40	178,069.90	174,680.10	186,984.30	185,686.00	184,416.60
K_2_	186,759.00	196,260.70	188,842.70	190,879.10	184,551.30	186,722.60
K_3_	180,705.60	183,452.40	186,868.40	182,047.50	178,973.00	184,411.10
K_4_	180,690.70	183,220.20	184,428.20	179,484.30	185,761.20	181,552.00
K_5_	182,774.60	179,137.10	185,320.90	180,745.10	185,168.80	183,038.00
k_1_	37,842.08	35,613.98	34,936.02	37,396.86	37,137.20	36,883.32
k_2_	37,351.80	39,252.14	37,768.54	38,175.82	36,910.26	37,344.52
k_3_	36,141.12	36,690.48	37,373.68	36,409.50	35,794.60	36,882.22
k_4_	36,138.14	36,644.04	36,885.64	35,896.86	37,152.24	36,310.40
k_5_	36,554.92	35,827.42	37,064.18	36,149.02	37,033.76	36,607.60
Rank3	1703.94	3638.16	2832.52	2278.96	1357.64	1034.12
R	4	1	2	3	5	6

**Table 7:** The final optimized parameters for AA-6082-T6.

	$\mu_{1}$	$\mu_{2}$	$\mu_{3}$	$k_{\omega }$	$f_{0}$	$f_{c}$
The original	0	0.4	0	0.5	0.008	0.2
The final	0	0.4	0	0.6	0.009	0.2
